# The Cytotoxic Effect of Copper (II) Complexes with Halogenated 1,3-Disubstituted Arylthioureas on Cancer and Bacterial Cells

**DOI:** 10.3390/ijms222111415

**Published:** 2021-10-22

**Authors:** Alicja Chrzanowska, Aleksandra Drzewiecka-Antonik, Katarzyna Dobrzyńska, Joanna Stefańska, Piotr Pietrzyk, Marta Struga, Anna Bielenica

**Affiliations:** 1 Chair and Department of Biochemistry, Medical University of Warsaw, 02-097 Warszawa, Poland; achrzanowska@wum.edu.pl (A.C.); mstruga@wum.edu.pl (M.S.); 2 Laboratory of X-ray and Electron Microscopy Research, Institute of Physics, Polish Academy of Sciences, 02-668 Warszawa, Poland; adrzew@ifpan.edu.pl; 3 Students’ Scientific Society at Department of Pharmaceutical Microbiology, Faculty of Pharmacy, Warsaw Medical University, 02-097 Warszawa, Poland; k-dobrzynska@o2.pl; 4 Department of Pharmaceutical Microbiology, Centre for Preclinical Research, Medical University of Warsaw, 02-097 Warszawa, Poland; jstefanska@wum.edu.pl; 5 Inorganic Chemistry Department, Faculty of Chemistry, Jagiellonian University, 30-387 Kraków, Poland; pietrzyk@chemia.uj.edu.pl

**Keywords:** copper (II) complexes, thiourea, cytotoxic activity, proteome analysis, antimicrobial activity

## Abstract

A series of eight copper (II) complexes with 3-(4-chloro-3-nitrophenyl)thiourea were designed and synthesized. The cytotoxic activity of all compounds was assessed in three human cancer cell lines (SW480, SW620, PC3) and human normal keratinocytes (HaCaT). The complexes **1**, **3**, **5**, **7** and **8** were cytotoxic to the studied tumor cells in the low micromolar range, without affecting the normal cells. The complexes **1**, **3**, **7** and **8** induced lactate dehydrogenase (LDH) release in all cancer cell lines, but not in the HaCaT cells. They provoked early apoptosis in pathological cells, especially in SW480 and PC3 cells. The ability of compounds **1**, **3**, **7** and **8** to diminish interleukin-6 (IL-6) concentration in a cell was established. For the first time, the influence of the most promising Cu (II) complexes on intensities of detoxifying and reactive oxygen species (ROS) scavenging the enzymes of tumor cells was studied. The cytotoxic effect of all copper (II) conjugates against standard and hospital bacterial strains was also proved.

## 1. Introduction

Enzymes incorporate transition metal cofactors to perform a wide range of metabolic reactions. Among metal cations, copper (II) ions play an essential function in the human organism, being a structural component of several enzymatic proteins, such as ascorbate oxidase, ceruloplasmin, amine oxidase, cytochrome C oxidase, nitrite reductase and superoxide dismutase Cu–Zn (SODC) [1,2]. Complexes of organic chelators with Cu (II) have been receiving increased attention due to their potential biomedical applications, such as cytotoxic or antibacterial activities. Within a group of acylthiourea ligands, those containing electron-withdrawing chloro- and nitro-substituents were effective against adenocarcinoma cell lines [3]. Intercalating properties of N-(2-hydroxyethyl)-N′-benzoylthiourea-Cu (II) complexes with strong deoxyribonucleic acid (DNA) binding and cleavage abilities were denoted [4]. Chelates with orthophenanthroline units studied by Pivetta et al. exerted high cytotoxic affect against acute leukemia and other carcinomas [5]. Moreover, a wide antibacterial profile of copper-based thiourea coordinates of 4-azatricyclo-3,5-dione [6] and 3-(trifluoromethyl)aniline was also studied. Their inhibitory effect on bacterial topoisomerases was also established [7].

Requirements for safer, more active and more selective chemotherapeutics have stimulated the search for other metal-based antitumor candidates. First of all, investigations in the treatment of cancer diseases are oriented to synthesize new analogues of cisplatin, a platinum (II) coordinate and chemotherapeutic drug effective against various types of tumors. Recently published Pd (II) and Pt (II) complexes of *N*-allylthioureas containing morpholine and *tert*-butylamine moieties showed proapoptotic and cytostatic influence on HeLa (Henrietta Lacks) cell culture stronger or comparable with cisplatin [8]. Antitumor properties of structurally similar coordinates based on pyrazol-3-ylpyridine fragments were connected with their inducing effect on cell accumulations in the S phase (HeLa cells) and a reduction of cell subpopulations in G_2_/M and G_0_/G_1_ phases (pancreatic cancer cells) [9]. Among tested palladium complexes of N, N-disubstituted thioureas, those endowed with furoyl and ethyl groups were the most effective against human breast cancer cells, as compared to the platinum-derived standard drug [10]. On the other hand, Marverti et al. [11] introduced a series of thiourea-Pd (II) compounds that acted as productive growth inhibitors of cisplatin-sensitive ovarian cancer cell lines and their resistant counterparts. Coordinates altered malignant cell metabolism via inhibition of thymidylate synthase and dihydrofolate reductase expression. Interestingly, photoactivated platinum (IV) azide dipyridine complexes bonded strongly to the nuclear DNA of tumor cells and blocked ribonucleic acid (RNA) polymerase II more efficiently than conventional adducts of cisplatin [12]. Platinum (II)-acridine hybrids linked to the amidine group [13] have shown inhibition of lung cancer cell proliferation and a promising antitumor potency in mouse xenograft models. In contrast, cationic gold (I) complexes of thiourea containing an acridine core are devoid of cytotoxic properties. However, they exert remarkable activity against *M. tuberculosis* [14]. Gold (I) complexes of cyclic disubstituted 4-chlorophenylthiourea ligand severely affected mitochondrial respiration by inhibition of complexes II and IV of the respiratory chain and induced mitochondrial swelling, resulting in enhanced permeability of a membrane and its declined fluidity [15]. An alternative class of anticancer drugs has been identified within ruthenium (II)–arene compounds. The Ru (II)-acylthiourea organometallic complexes were found to be significantly cytotoxic towards lung [16,17,18], prostate [16] and breast [18] tumor cell cultures, inducing their apoptosis and reducing migration by interaction with human serum albumin. What is more, other results showed that silver (I) chelates of 2-benzimidazolylurea mediate a strong cytotoxic response to the tested breast cancer cell lines (MCF-7) and normal cells, but with better parameters than metallodrug cisplatin [19].

Reactive oxygen species (ROS), produced in the mitochondria, peroxisomes and endoplasmic reticulum, include highly reactive species such as hydroxyl and superoxide free radicals, singlet oxygen or less reactive hydrogen peroxide molecules. Regulation of ROS levels is crucial for cellular life, cell proliferation and differentiation. Under oxidative stress states, accumulation of ROS causes the damage of proteins, lipids and DNA, which contributes to carcinogenesis [20]. The expanded scavenging system of a cell is based on superoxide dismutases (SODs), glutathione peroxidase (GPX), glutathione reductase (GR), peroxiredoxins (PRDXs), thioredoxin and catalase (CAT), which neutralize reactive species or recover antioxidants to their reduced state [21]. In comparison with normal cells, an extent of ROS in cancer counterparts is higher, as they keep an improved antioxidant system [22]. What is more, expanded levels of GPX, CAT, glutathione and thioredoxin proteins are correlated with tumor aggression and its resistance to chemotherapy [22]. It was proved that a decline of ROS-scavenging capacity leads to apoptosis of malignant cells, especially when it is used in combination with ROS-increasing chemotherapeutics, such as 5-fluorouracil (therapy of colon cancer), celecoxib (prostate tumor) or doxorubicin (breast, liver cancers) [21,23,24,25,26]. Numerous organic complexes with transition metals are reported to induce autophagy and/or apoptosis by regulation of ROS. This group includes complexes of gold (I) with thiourea [15,27], ruthenium (II) coordinates with β-carboline [28], copper (II) complexes with 2-hydroxy-1-naphthaldehyde [29] and with the derivative of bipyridine-acetylacetonate (Casiopeina III-ia) [30]. The antioxidant abilities of coordinates evaluated by the diphenyl-2,2-picrylhydrazyl/ N, N-diethyl-p-phenylenediamine (DPPH/DPD) method were also confirmed for ruthenium (III) chelates with 1-ethyl-3-phenylthiourea ligands [31] and copper (II) complexes with biuret or urea [32], as well as with auxin [33] or N-ethylpiperazine [34].

As a part of our research on developing biologically active 1,3-disubstituted thioureas [7,35,36,37], we have synthesized a series of halogenated copper (II) complexes of (4-chloro-3-nitrophenyl)thiourea and reported their cytotoxic, prooxidant and antimicrobial properties.

## 2. Results and Discussion

### 2.1. Synthesis of Complexes

**Figure 1:**
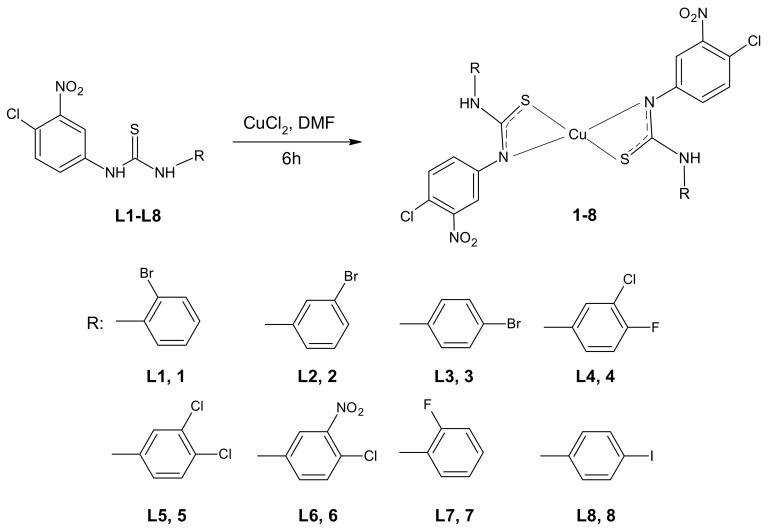
Scheme of the complexation reaction of the respective arylthiourea ligands **L1**–**L8** with proposed molecular structures of complexes **1**–**8** (DMF—dimethylformamide).

Thiourea–Cu (II) coordination compounds **1**–**8** were prepared by the reaction of copper (II) chloride with 3-(4-chloro-3-nitrophenyl)thiourea compounds (Figure 1). The complexes were obtained with satisfactory yields (45–61%). The identification of the parent ligands **L1**–**L8**, using nuclear magnetic resonance (NMR) and mass spectroscopy, was described previously [37,38]. The derivatives chosen for complexation reactions came from a series of mono- (**L4**–**L6**) or di-halogenated (**L1**–**L3**, **L7**, **L8**) 1-phenylthioureas with the most promising bioactivity. The presented selection of the phenyl ring substituents in their structures allow for the analysis of the influence of the substitution isomerism, as well as the impact of electron-withdrawing elements on the biological properties of newly synthesized metal–organic compounds.

### 2.2. Structural Characterization of Complexes

The complexes were initially characterized by elemental analysis and infrared spectroscopy. During all complexation reactions, disubstituted thiourea acts as bidentate ligand (L), forming a hydrated complex of the type CuL_2_. In order to determine the geometry of metal–organic ligand complexes, ultraviolet–visible (UV–Vis) and electron paramagnetic resonance (EPR) spectroscopies have been applied.

**Figure 2:**
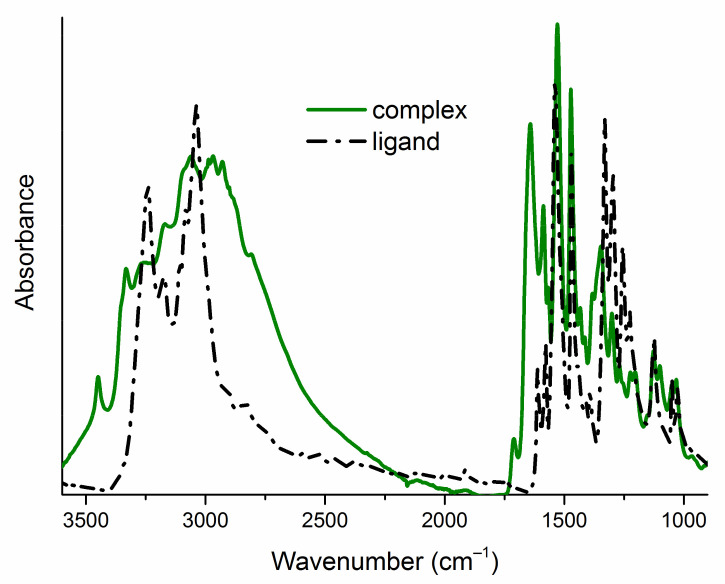
Attenuated total reflection infrared (ATR-IR) spectra of one exemplary pair of a free ligand (**L5**) and a complex (**5**) in the range of 3600–900 cm^–1^. ATR-IR spectra of all studied compounds are gathered in the Supplementary Materials (Figure S1).

The infrared spectra of ligands (**L1**–**L8**) exhibit a broad band with several maxima in the high-energy part (Figure 2), corresponding to the stretching vibrations of the N–H and C–H groups. In the spectra of Cu (II) complexes (**1**–**8**), this band widens (Figure 2), which confirms the presence of water molecules in the product of the complexation reactions. The molecules of the initial ligands (**L1**–**L8**) and their complexes (**1**–**8**) contain two N–H groups (Figure 1), which are reflected in two infrared absorption bands. These bands are in different positions in the spectra of a pair of free and bonded ligands (Figure 2, Table 1). This confirms deprotonation of one N-atom from the ligand’s molecules and suggests different intermolecular interactions (N–H... X; X=O, Br, Cl, F, I hydrogen bonding patterns) in the crystal structure of free and bonded thioureas. The region of 1600–1400 cm^−1^ in the spectra of ligands (**L1**–**L8**) is dominated by the stretching vibrations of the C–C and C–N bonds. In the spectra of complexes (**1**–**8**), this feature is extended towards the higher frequencies (around 1700 cm^−1^, Figure 2) as the consequence of increased intensities of asymmetric ring stretching modes within the molecules of complexes [7]. The spectral ranges: 1550–1500 cm^−1^ (NO_2_ asymmetric stretching vibrations), 1360–1330 cm^−1^ (NO_2_ symmetric stretching vibrations) and 1240–1040 cm^−1^ (C–halogen stretching modes) are quite similar for parent ligands and their Cu (II) complexes (Table 1), which excludes the interaction of the nitro group or the halogen atom with the metal cation. The bands corresponding to the C=S stretching vibrations are found around 1333 cm^−1^ and in the 854–832 cm^−1^ range in the uncomplexed ligand’s spectra (Table 1). In the spectra of complexes, they are red shifted and observed at 1313–1300 cm^−1^ and 785–771 cm^−1^ regions (Table 1). This indicates that the ligands coordinate to the Cu (II) through the sulfur atom [39,40,41].

**Figure 3:**
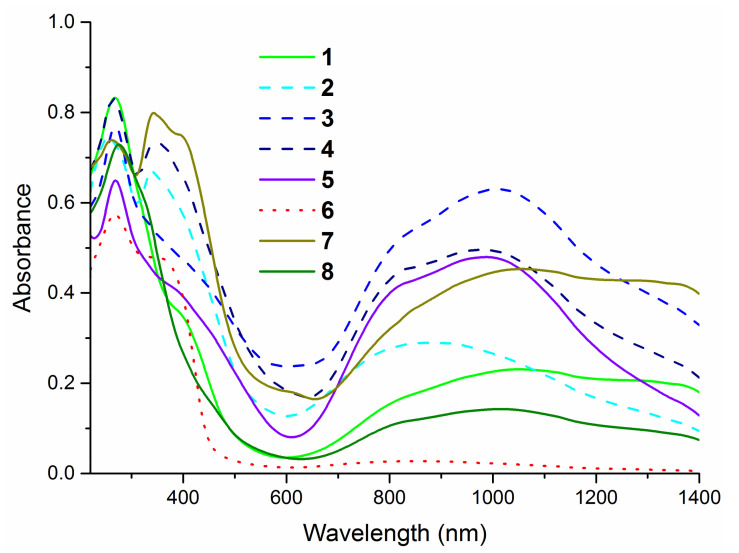
Solid state ultraviolet–visible (UV–Vis) spectra of complexes in the range of 220–1400 nm.

The electronic spectra of the complexes (Figure 3) exhibit the intense absorption bands in the region 230–300 nm corresponding to the π→π* and n→π* intra-ligand transitions. The presence of S→Cu (II) ligand-to-metal charge transfer (LMCT band) around 400 nm is additional evidence of the coordination of thiourea ligands to the metal cation via sulfur atom [39,40,42]. All presented compounds show the *d-d* bands in a wide region 650–1400 nm, which confirms the formation of four-coordinate complexes with copper on +2 oxidation state [41].

**Figure 4:**
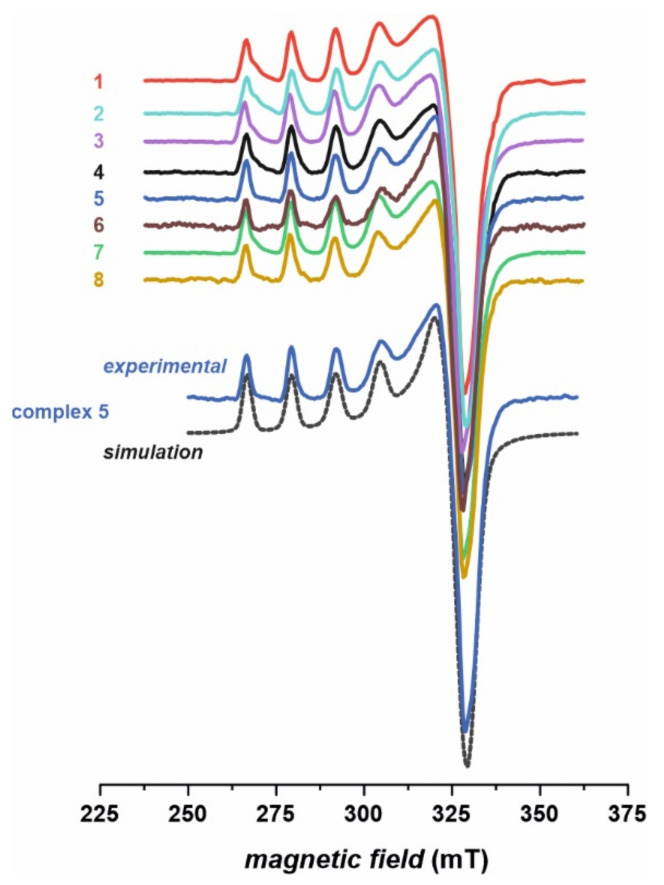
Frozen solution (77 K) X-band electron paramagnetic resonance (EPR) spectra of complexes **1**–**8**, along with exemplary computer simulation of spectrum for the complex **5**.

EPR spectra of the investigated complexes **1**–**8** (Figure 4) are in agreement with the conjectures drawn based on the infrared (IR) and UV–Vis results. The obtained spectra indicate clearly the divalent state of copper upon complexation and are very characteristic of typical Cu (II) complexes. The signals were simulated assuming rhombic symmetry with the *z* component (parallel) split into four lines due to the hyperfine interaction between the unpaired electron and nuclear magnetic moment of copper (*I* = 3/2, ^63,65^Cu). The splitting of the perpendicular line (*x* and *y* components) remains unresolved, yet it was necessary for obtaining the correct shape of the simulated spectra. Slight rhombicity of the spectra with *g*_zz_ >> *g*_xx_ > *g*_yy_ confirms dominant contribution of a single d-orbital (3d_x_^2^_–y_^2^) in the semi-occupied molecular orbital. Exemplary parameters obtained for compound **5** are: *g*_xx_ = 2.086, *g*_yy_ = 2.054, *g*_zz_ = 2.357, *A*_xx_ and *A*_yy_ unresolved, |*A*_zz_| = 12.8 mT. Similar parameters were recently reported for Cu (II) thiourea complexes [6,7]. The EPR parameters obtained for other complexes are very similar (12.6 mT < |*A*_zz_| < 12.8 mT, 2.353 < *g*_zz_ < 2.358), which strongly indicates very similar coordination around the copper center. It is in agreement with the molecular structure of the complexes (with two thiourea ligands chelated to the Cu (II) cation via deprotonated N and thiocarbonyl S atoms), which differ only in the substituents of thiourea ligands (Figure 1). Such modification has only a minute effect on the first coordination sphere of copper, to which continuous wave EPR is sensitive. No features characteristic of Cu dimer in solution were observed, e.g., a forbidden magnetic dipolar transition at half-field with *g* = 4 value.

The molecular structure proposed for compounds **1**–**8**, in which the Cu (II) cation is the part of two four-membered rings (Cu-S-C-N, see Figure 1), is quite unusual for copper. However, this type of coordination was reported for one crystal structure determined by Singh et al. [43]. They found Cu (II) ions being chelated by two thiourea ligands in a trans manner and the distorted square-planar geometry of the complex was stabilized by electron delocalization in chelate rings. Moreover, such coordination was postulated by some of the authors of the current study for another series of Cu (II) complexes, namely for 3-(trifluoromethyl)phenylthiourea derivatives [7] and thioureas containing tricyclic imide’s part [6,41]. Structural characterization of those complexes, possibly owing to the combination of several experimental laboratory and synchrotron techniques (including X-ray absorption spectroscopy) and molecular modelling, indicated that 1,3-disubstituted thiourea ligands coordinate to Cu (II) cation in bidentate fashion through S and N atoms, forming a four membered 1,3-N, S chelate ring (as presented in Figure 1 for compounds **1**–**8**).

### 2.3. Biological Studies

#### 2.3.1. Anticancer Activity

##### MTT Assay

In vitro cytotoxicity studies of synthesized thiourea complexes **1**–**8** were assessed by standard 3-(4,5-dimethylthiazol-2-yl)-2,5-diphenyltetrazolium bromide (MTT) bioassay in different cancer cells at 72 h of drug exposure (Table 2). The ability of compounds to inhibit cell proliferation was established by means of their half-maximal inhibitory concentration (IC_50_) values towards the human tumor cell lines, such as SW480 (primary colon cancer), SW620 (metastatic colon cancer) and PC3 (metastatic prostate cancer), as well as against the non-tumor cell line HaCaT (immortal keratinocytes).

The 1,3-disubstituted arylthiourea complexes **1**, **3**, **5**, **7** and **8** were cytotoxic to studied cancer cells at concentration ≤ 10 µM. The monohalogen derivatives **1** and **8** showed cytotoxic activity in the low micromolar range in all tested pathological cell lines. Their IC_50_ values ranged from 3.3 ± 0.2 to 9.7 ± 0.1 µM (compound **1**) and between 3.9 ± 0.8 and 17.8 ± 1.3 µM for compound **8**. In particular, they both were strongly potent towards SW480 cells (at 3.9 ± 0.8 and 4.7 ± 0.3 µM), whereas derivative **1** was against SW620 (at 3.3 ± 0.2 µM) and derivative **8** towards the PC3 cell line (with IC_50_ of 4.3 ± 0.5 µM).

The 4-bromophenyl compound (**3**) exerted remarkable growth-inhibiting activity mainly against PC3 cells (8.8 ± 0.8 µM). On the other hand, the 2-fluorophenyl-containing molecule **7** exerted the highest IC_50_ values for SW480 and PC3 cell lines (9.1 ± 0.8 and 10.8 ± 1.3 µM, respectively). Dichlorophenyl derivative **5** was potent against SW620 cancer cells at 10.8 ± 2.6 µM.

In general, the PC3 cell line was the most sensitive to the presence of new complexes, and substances **1**, **3** and **8** appeared the most effective in the inhibition of their rise. The same set of compounds acted the strongest against primary cancerous SW480 cells. In contrast, for substances **1**, **5** and **7**, the lowest IC_50_ indexes for metastatic SW620 cells were denoted. The cytotoxicity of other tested derivatives (**2**, **4**, **6**) towards pathological cell lines was moderate, with IC_50_ values from 19.2 ± 1.1 to 26.8 ± 2.3 µM.

The studied thiourea complexes were non-cytotoxic against normal HaCaT cell lines (IC_50_ ˃ 100 µM). The highest selectivity indexes (SI) were observed for the most promising derivatives: **1** (33.2 vs. SW620; 23.3 for SW480) and **8** (26.3 vs. SW480; 23.8 vs. PC3 cells). The selectivity factors of other complexes **2**–**7** were in the range of 4.6–8.9 (SW480), 5.3–15.2 (SW620) and 5.2–14.4 (PC3 cell line). These indexes were incomparably greater than those of the reference doxorubicin and cisplatin (SI between 0.4 and 1.1), which proves a low toxicity of tested complexes towards health cells.

As compared to cisplatin, the cytotoxic properties of the synthesized diarylthiourea derivatives were considerably stronger. Complexes **1**, **3**, **5**, **7** and **8** were 1.2–3.1-fold more active against human PC3 cell lines. Additionally, both studied colon cancer cell lines appeared to be 2–2.7 times more sensitive to the presence of coordination compounds **1** and **8** than cisplatin, one of the most frequently used chemotherapy drugs.

The present study reveals that monohalogen substituted phenylthiourea complexes (**1**–**3**, **7**, **8**) possess stronger cytotoxic properties in human cancer cell lines, when compared to disubstituted derivatives (**4**–**6**). The moderately electronegative atom, such as bromine or iodide at the ortho (**1**,**7**) or para (**3**, **8**) position of the aromatic ring, was responsible for enhanced cytotoxicity in the MTT model. The location of an element at meta position (**2**) resulted in a severalfold reduction in anticancer activity. Among disubstituted halogen coordinates, the 3,4-dichlorophenyl derivative (**5**) appeared to be the most effective towards SW620 and PC3 cells. The substitution of the benzene with chlorine and nitro group together provided a less promising drug candidate (**6**), with IC_50_ at least 20.3 ± 3.1 µM against all tested cancer cells. Comparing pairs **7** (2-fluorophenyl-) and **4** (4-fluoro-3-chlorophenylthiourea), an introduction of the second halogen to the molecule led to the twofold decrease in cytotoxic activity vs. metastatic SW620 and PC3 cell lines. In fact, the location of two different groups in both third and fourth position of the ring was not beneficial on biological activity (**4**,**6**). Only 3,4-dichlorophenyl complex (**5**) restrained the potency in the low micromolar range, as compared to its monohalogen analogs **3** and **7**. To sum up, the phenyl ring substituents can be arranged in order of their decreasing impact on bioactivity as follows: 2-bromo > 4-iodo > 2-fluoro > 4-bromo > 3,4-dichloro >> 3-chloro-4-fluoro > 3-bromo > 3-nitro-4-chloro. It is worth noting that all of them were at least 350 times less toxic for normal HaCaT cell lines than reference doxorubicin.

As reported, the cytotoxicity of standard copper (II) chloride towards cancer cells was negligible in comparison with thiourea derivatives. Its C_50_ ranged between 96.3 and 109.4 µM, and the salt was nontoxic for normal cells (IC_50_ >100 µM). DMF, an organic solvent that contaminates synthesized complexes, has already been tested on various cells and appears to be nontoxic at the concentrations used in our synthesis (see in the Supplementary Material section). Considering an influence of starting ligands **L1**–**L8** on studied cell lines, the derivative **L5**, at the concentration 8 µM, caused an approximately 20% decrease in HaCaT cell viability, which indicated its weak cytotoxic impact against normal cells [36]. In contrast, new complexes are devoid of visible influence on keratinocytes. In preliminary tests published previously, ligands **L1**, **L3**–**L5** and **L8** influenced the growth of other cancer cells, such as human leukemia and solid (melanoma, prostate) tumors, as well as normal tissue foreskin fibroblasts [36]. These results encouraged us to proceed with deeper investigations on the cytotoxicity mechanism of complexes **1**–**8**.

##### LDH Assay

The lactate dehydrogenase (LDH) release assay was used to determine the level of plasma membrane damage of the most distinctive derivatives **1**, **3**, **7**, **8**. They were studied at concentrations of 10–60 μM against cancer cell lines and 60–120 μM towards normal HaCaT cells.

**Figure 5:**
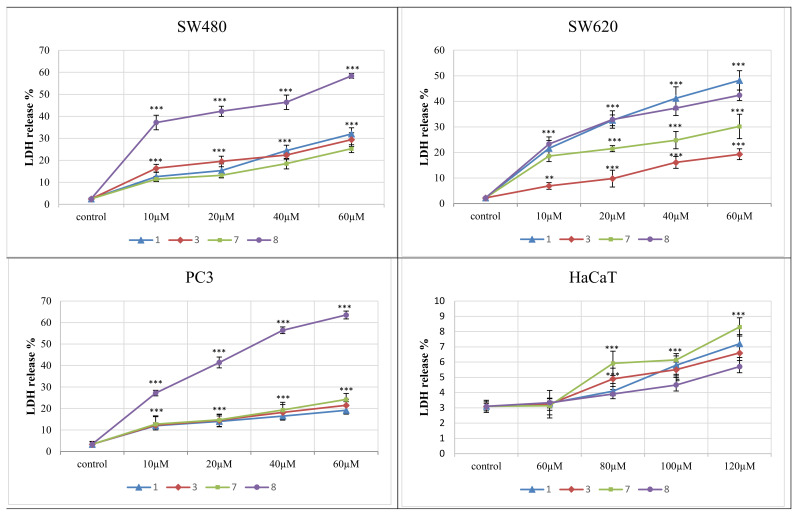
Lactate dehydrogenase (LDH) release as a marker of cell death in the SW480, SW620, PC3 and HaCaT cells, treated for 72 h with different concentrations of compounds **1**, **3**, **7** and **8**. LDH release in the HaCaT was analyzed after treatment with higher doses of compounds. *** *p* ≤ 0.001 as compared to the control.

The LDH release curve results (Figure 5) demonstrated that the applied amounts of complexes did not affect the normal keratinocytes viability, as the secretion of LDH in their presence varied from 1.3% to 5.7%. The 4-iodophenylthiourea complex **8** exerted the highest cytotoxicity against all three tumor cell lines. This effect was the most evident in PC3 and SW480 cells, compared to SW620. When used at 60 μM, the derivative **8** achieved 64% LDH release in PC3 cells and 58% in SW480 cell lines. LDH secretion in these cells accounted for other complexes, used at the highest concentrations, ranging from 19% to 32%. The compound **8**, applied in a dose of 40 and 20 μM, also expressed an increased response against SW480 cells (LDH release was 46.4% and 42%, respectively). Similarly, the LDH leakage induced in PC3 cells by this derivative used at lower concentrations differed from 56.4% (40 μM) to 27.2 % (10 μM). On the other hand, the number of lysed SW620 cells evoked by the presence of the 2-bromophenylthiourea complex **1** ranged from 48% (at 60 μM) to 22% (10 μM applied), and incubation of these cell lines with the derivative **8** expressed similar enzyme releases of 42.4% and 23%, respectively. 2-fluorophenyl compound **7**, studied at its highest dose, was less cytotoxic and achieved 30.2% LDH secretion.

The reference cancer chemotherapeutic doxorubicin showed very high LDH release in all studied cancer cell lines, even when applied in lower doses. The LDH percentage for this compound at 1.5 μM varied from 70% to 98%. However, its toxicity against normal HaCaT cells was significantly higher than examined complexes and accounted for 80%.

The data gained by the LDH activity assay are in an agreement with the results obtained for derivatives **1**, **3**, **7** and **8** by the MTT method.

##### Apoptotic Activity

**Figure 6:**
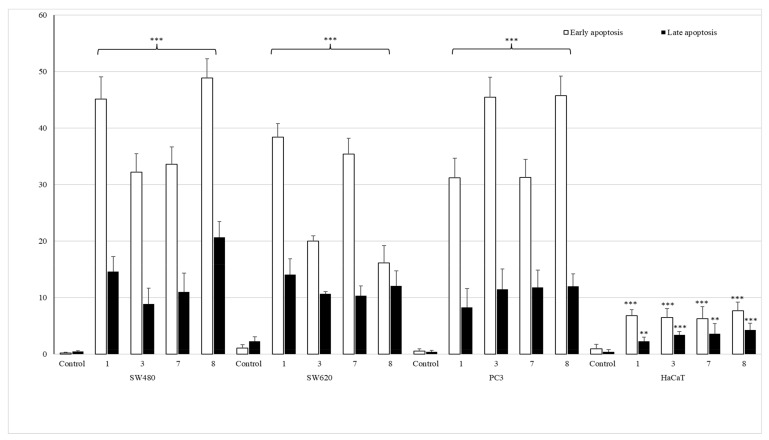
The effect of complexes **1**, **3**, **7** and **8** on early and late apoptosis, or necrosis in SW480, SW620, PC3 and HaCaT cells. Cells were incubated for 72 h with tested compounds used in their IC_50_ concentrations. Then, cells were harvested, stained with annexin V-FITC and PI (fluorescein isothiocyanate and propidium iodide) and analyzed using flow cytometry. *** *p* ≤ 0.001, ** *p* ≤ 0.01 as compared to the control.

To establish the anticancer mechanism of activity, SW480, SW620 and PC3 cells were incubated for 72 h in the presence of the most promising monohalogeno complexes **1**, **3**, **7** and **8**, after which annexin content was measured by flow cytometry analysis. The apoptotic effect is shown in Figure 6 and Figure S2.

All studied compounds, applied in their IC_50_ doses, induced early apoptosis in pathological cells, especially in SW480 and PC3 cell lines, as compared to untreated cancerous controls. Derivatives of 2-bromophenyl- (**1**) and 4-iodophenylthiourea (**8**) revealed the most significant early-apoptosis-activating effect in primary colon cancer cell lines (45.18% ± 3.9% and 48.9% ± 3.4%, respectively). The influence of complexes bearing 4-bromophenyl (**3**) and 2-fluorophenyl (**7**) moieties in these cells was not so evident and did not exceed 40%. The most potent activators of apoptosis in metastatic PC3 cells were both para-substituted coordination compounds **3** and **8**, as they promoted the process in 45.5% ± 3.5% and 45.7% ± 3.3% of cells. On the other hand, complexes containing a halogen atom in ortho position of the benzene ring (**1** and **7**) induced the early apoptosis in one third of tested PC3 cells. The similar pro-apoptotic inducing effect of these two compounds was observed in metastatic colon cancer cells (38.4% ± 2.4% and 35.4% ± 2.8%). The incubation of these cells with derivatives **3** or **8** increased early apoptosis in only approximately 20%.

The studied set of thiourea complexes did not considerably promote the process of late apoptosis of cells, as compared to controls. Only the most cytotoxic derivative **8** had the late apoptosis activating property, in 20.7% ± 2.8% of SW480 cells. It is also worth noting that tested coordination compounds **1**, **3**, **7** and **8** did not affect the level of early/late apoptosis in normal human keratinocytes (HaCaT), giving the result from 1.6% to 9.2% only. 

In addition, conducted studies revealed that the referential doxorubicin was only a necrosis inducer. The number of SW480, SW620 and PC3 cells at the necrotic stage was 86%, 51% and 50%, respectively. Only in the case of PC3 cells, it induced late apoptosis as well (23%). HaCaT cells were considerably sensitive to doxorubicin, which produced a necrotic stage in 42% of cells, without affecting early and late apoptosis.

The obtained data comply with IC_50_ results found for mentioned cancer cells, particularly indicating the pro-apoptotic promoting role of complexes **1** and **8** in SW480 cells, as well as substances **3** and **8** in the prostate cancer cell line.

##### IL-6 Assay

Human interleukin-6 (IL-6) is a pro-inflammatory cytokine, involved in numerous biological processes such as inflammation, cell growth, apoptosis, aging or bone remodeling. The ability of compounds to diminish IL-6 concentration in a cell, as a measure of their anti-inflammatory properties, was established for the most cytotoxic copper (II) complexes of thiourea derivatives (**1**, **3**, **7**, **8**).

**Figure 7:**
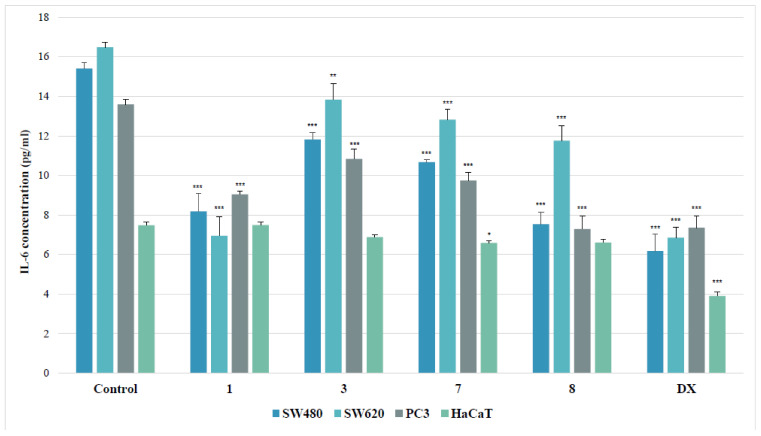
Effects of thiourea complexes **1**, **3**, **7** and **8** on IL-6 levels, measured by enzyme-linked immunosorbent assay (ELISA) test. Data are expressed as the mean ± SD from three independent experiments performed in triplicate. *** *p* ≤ 0.001, ** *p* ≤ 0.01, * *p* ≤ 0.05, as compared to the control. DX (doxorubicin) is the reference compound.

As shown in Figure 7, tested derivatives applied in their IC_50_ doses inhibited interleukin release in all evaluated cancer cell lines. Among them, the SW480 line was the most susceptible for the inhibition of the IL-6 secretion, elicited by the presence of evaluated substances. The 4-iodophenyl-containing complex (**8**) diminished the IL-6 level in these cells twofold. Similarly, the treatment of the primary colon cancer cells with 2-bromophenylyhiourea coordinate (**1**) reduced the cytokine amount 1.9 times. However, the other complexes (**3** and **7**) decreased that level not more than by 30%, as compared to the controls. The effectiveness of the complex **1** in metastatic SW620 cells equaled to the referential doxorubicin. Both substances inhibited the concentration of IL-6 2.4 times, while other tested derivatives diminished its secretion by an average of 23%. The strongest effect in PC3 cells was denoted for the 4-iodophenylthiourea complex (**8**), which, just as doxorubicin, diminished the IL-6 secretion almost twofold. On the other hand, the incubation of PC3 cells with the 2-bromophenyl compound (**1**) lowered the IL-6 concentration 1.5 times, with weaker effect of other complexes (**3** and **7**). 

##### Proteomic Analysis of Antioxidant and Detoxifying Enzymes

To evaluate the impact of the tested compounds on antioxidant status, they were screened for their influence on intensities of selected detoxifying and ROS-scavenging enzymes, occurring in human cancer cell lines such as glutathione S-transferase (GST), glutathione reductase (GR), superoxide dismutase (SOD) and peroxiredoxin (PRDX) (Table 3 and Table S1).

All tested complex compounds, applied in their IC_50_ concentrations, reduced intensities of colon and prostate cancer enzymatic proteins, in most cases by at least 30% of a control. The types of studied thiourea terminal moieties can be arranged in order of their decreasing influence, as shown: 2-fluoro- (**7**), 4-bromo- (**3**), 4-iodo- (**8**) and 2-bromophenyl (**1**). The most susceptible for the presence of tested coordinates were the proteins of metastatic PC3 cells. The level of all tested enzymes in the PC3 cell was diminished, while simultaneously superoxide dismutase [Cu-Zn] (SODC), mitochondrial superoxide dismutase (Mn) (SODM) and the majority of PRDXs belonged to the most sensitive. The 2-fluorophenyl derivative (**7**) reduced the intensity of 12 prostatic enzymes by 20.8-87.6%. Among them, both types of SOD and mitochondrial PRDX3 were the most vulnerable; their intensities were reduced by at least 75%. The compound **3** diminished the amount of all tested proteins in the PC3 cells by 29.4–69.1%, affecting most significantly the levels of both SODs. Similarly, after treatment of the cells with the complex **8**, the amount of 12 enzymes was reduced by 31.5–75.2%, including glutathione S-transferase A1 (GSTA1), SODs, PRDX3 and PRDX4, which intensities equaled about 30% of a control. After incubation with the coordinate **1**, quantities of SODM and PRDX5 were diminished up to 29% of the initial level.

On the other hand, GSTA1, glutathione S-transferase P (GSTP1) and PRDX3 of metastatic SW620 cells were the most susceptible to the treatment with copper (II) complexes. The derivative **7** appeared to be the most powerful. It reduced intensities of all tested colon proteins by 32.5–63.9%, most heavily both GSTs (up to 63.6%) and SODs (55.3%). Other analogs (**1**, **3**, **8**) affected considerably mainly on GSTA1, leading to a 45.7% (compound **8**) and 54.8% (complex **3**) decrease in the amount of this protein, respectively.

The impact of the tested compounds on enzymes of the primary SW480 cells was also evident, but weaker than that observed for both metastatic lines. The quantitative changes affected mainly GSTA1, SODC and PRDX4. The derivative **7** exerted the strongest effect, reducing their intensities by 54.9%, 59.4% and 41.7%, respectively. Additionally, it diminished the amount of five other proteins by 28.4% or more. Complexes **1** and **3** acted similarly to their analogs, but they also affected smaller group of enzymes, most significantly mitochondrial glutathione reductase (GSHR) (compound **1**) and PRDX4 (compound **3**). The thiourea coordination compound **8** had the greatest relevance for GSTA1, giving 47.6% inhibition of its intensity.

Obtained results confirmed the cytotoxic effect of tested complexes by the decreasing of antioxidant influence on cytoplasmic and mitochondrial proteins of malignant cells, especially observed for derivatives **7** and **3**.

According to the literature data, there are few evidences that suggest GSTs function in the cancer development and resistance of colon and prostate tumor cells to anticancer agents. Colon cancers reflected elevated levels of GST expression compared with normal mucosa [44]. It was observed that GST overexpression was related to a doxorubicin-resistance phenomenon [45]. Similarly, the high level of glutathione-S-transferase pi (GST-pi) was detected in prostate cancer cells, and it contributed to the development of drug resistance and a significant increase in the cell proliferation rate of androgen independent PC3 cells [46].

Likewise, an increased GR expression and activity was shown in colon tumors, where it was involved in cellular defense against ROS by producing a reduced form of GSH. This activity may favor tumor development [47]. It was also shown that this enzyme may protect PC3 cells under persistent ROS production induced by radiotherapy and chemotherapy. Therefore, GR activity depletion could lead to the intensified H_2_O_2_ toxicity in metastatic prostate cancer cells [48].

There are contradictory reports indicating the function of SOD enzymes on primary tumor proliferation and metastasis activity. It was shown that overexpression of SOD could increase cell differentiation, decrease proliferation and turn back a malignant phenotype [49]. Other studies demonstrated that high SOD expression correlates with colon tumor aggressiveness and with resistance to cytotoxic drugs and radiotherapy [50]. The loss of SOD1 expression by siRNA knockdown significantly increased prostate cell sensitivity to cytotoxic agents, confirming SOD1 participation in cellular response and resistance [51]. Some evidence has indicated SOD1 overexpression in cancers maintaining cellular ROS below the crucial threshold [52]. Suddenly, down-regulation of SOD2 itself was reported in breast cancer cell lines [53].

Beyond the PRDX family protein’s regulatory function on cytokine-induced hydrogen peroxide concentration, some members can act independently during their peroxidase activity on cell proliferation, differentiation, apoptosis and gene expression [54]. Recent studies highlight dual catalytic activities of PRDX1, because besides antioxidant activity it has a physiologically significant overoxidation site [55,56]. The positive PRDXI immunohistochemical staining was strongly associated with a poor response to neoadjuvant chemoradiotherapy and a worse prognosis in rectal cancer patients [57]. The PRDX3 protein in antiandrogen resistant prostate cancer cell lines is responsible for increased tolerance to oxidative stress and a lack of activation of pro-apoptotic pathways. Therefore, the knockdown of PRDX3 leads to an increased tendency to oxidative stress [58]. In vivo studies showed the up-regulation of both PRDX3 and PRDX4 in prostate tumors. It could be an attempt at adaptation of the cancer cells to the microenvironment in a way favorable for survival and proliferation rate, while maintaining their tumor’s aggressiveness [59].

#### 2.3.2. In Vitro Antimicrobial Activity

To assess the antimicrobial profile of coordination compounds **1**–**8**, we assembled a panel of isolates, which included Gram-positive and Gram-negative organisms of clinical importance, as well as strains of fungal pathogens. The synthesized derivatives displayed significant to weak inhibitory effects towards standard staphylococci, as shown in Table 4. Derivatives of 3-chloro-4-fluorophenyl- (**4**) and 4-iodophenylthiourea (**8**) appeared to be the most active, with MIC values of 4 µg/mL. Bromophenyl compounds (**1** and **3**) and 3-nitro-4-chlorophenyl-containing complex (**6**) moderately inhibited the growth of *S. aureus* isolates, at concentrations from 4 to 8 µg/mL. The least potent monohalogen derivatives **2**, **7** and dichlorophenyl complex **5** in doses of 16–32 µg/mL suppress the rise of staphylococcal rods. On the other hand, Gram-negative strains were weakly susceptible to the presence of tested thiourea coordinates (minimal inhibitory concentration, MIC ≥ 128 µg/mL). No evident antifungal properties were also observed; compounds **2**, **3**, **6** and **8**, applied only at a dose of 64 µg/mL, exerted growth inhibitory properties towards studied *Candida* species

On extended testing, a series of copper (II) complexes showed activity against 30 clinical methicillin-resistant strains of *S. aureus* (MRSA) and *S. epidermidis* (MRSE) (Table 5), and their potency against most of above-mentioned cocci was severalfold higher than the reference ciprofloxacin. The derivative **3**, possessing 4-bromophenyl substituent at the thiourea branch, exhibited its growth-inhibitory effect towards all evaluated hospital *S. aureus* rods with MIC of 4 µg/mL, whereas against most of *S. epidermidis* species at a dose of 4–8 µg/mL. The similar sensitivity of clinical isolates of *S. aureus* to the presence of 3-chloro-4-fluorophenyl complex (**4**) was observed. Monosubstituted compounds, containing bromine (**1**, **2**), fluorine (**7**) and iodide (**8**) on the phenyl ring, also possessed a moderate antibacterial potency (MIC 4–8 µg/mL). However, complexes **3**, **4**, **7** and **8** were 64 times more active towards *S. aureus* 572 and 481 strains than ciprofloxacin, while the bioactivity of coordinates **1**–**4**, **7** and **8** against six clinical *S. aureus* rods was 16–64 times stronger than the reference drug. Disubstituted derivatives **5** and **6** acted with lesser strength than their analogues incorporating one halogen in a molecule, but they were still 4–16 times more potent as compared to ciprofloxacin.

Most of the clinical strains of *S. epidermidis* were less susceptible to the presence of the synthesized complexes. However, derivatives **1**, **2**, **4**, **7** and **8** appeared to be 2–16 times more effective against *S. epidermidis* 424, 471, 511, 515 and 431–433 isolates than the reference chemotherapeutic. Similarly, weak antimicrobials, such as disubstituted compounds **5** and **6**, were still equally or more potent towards these cocci, as compared to ciprofloxacin.

To sum up, thiourea complexes incorporating a copper (II) ion expressed higher inhibitory growth properties against hospital than standard bacterial rods. What is more, their efficiency depended on the type of electronegative functionalities attached to the benzene ring. Derivatives incorporating 3-chloro-4-fluorophenyl- (**4**) and 4-iodophenyl substituents (**8**) showed the strongest effect towards both types of strains, while their 4-bromosubstituted analog (**3**) was powerful distinctly against clinical isolates. Ortho-substituted compounds, bearing bromine (**1**) or fluorine (**7**) atoms, shared a similar inhibitory activity when incubated with *Staphylococci*, but were weaker than the mentioned para-substituted. Comparing dihalogeno derivatives **4** and **5**, the replacement of the fluorine atom by chlorine led to a severalfold decrease of the antimicrobial potency. On the other hand, the compound possessing both chlorine and nitro group (**6**) was much more active against standard than hospital *S. aureus* species.

As proved by *Bacillus subtilis* rec-assay test, newly synthesized complexes **1**–**8** exerted no mutagenic and carcinogenic activities (Table S2). Thus, their antimicrobial effects were not linked with DNA-damaging potency.

As compared to results obtained for complexes, the antimicrobial activities of parental ligands were considerably higher [37]. MIC values of the most active ligands **L1**–**L5**, **L7** and **L8** against standard Staphylococcal strains ranged from 0.5 to 2 µg/mL. As expected, new copper (II) complexes were much more potent than copper (II) chloride itself (MIC ≥ 128 µg/mL for all strains). Although the complexation of halogen-containing 4-chloro-3-nitrophenylthioureas with copper (II) ion diminished their antibacterial properties [36,37], it incomparably increased their cytotoxicity towards various cancer cell lines, without influencing on normal keratinocytes.

Antimicrobial activity of the close analogs of the title complexes was previously described [6,7]. The formerly tested copper (II) complex of 1-(3-chloro-4-fluorophenyl)-3-[3-trifluoromethyl)phenylthiourea, an analog of the complex **4** described in this paper, exerted comparable growth-inhibitory activity towards standard bacterial strains (MIC 4–256 μg/mL) [7]. The same level of activities of both compounds was observed for clinical strains (MIC 4–8 μg/mL). However, for the derivative of 3,4-dichlorophenylthiourea, the observed differences in bioactivity were considerable. The complex of 3-(trifluoromethyl)phenylthiourea inhibited the growth of Staphylococcal strains at 2 μg, whereas the coordinate of 1-(4-chloro-3-nitrophenyl)-3-(3,4-dichlorophenyl)thiourea (**5**) was only at 32 μg/mL. Variabilities in antibacterial actions were even higher when hospital strains were considered. The 3-(trifluoromethyl)phenylthiourea analog was 16-64 times more potent than its 4-chloro-3-nitrophenylthiourea counterpart (**5**). On the other hand, copper (II) complexes with 2-bromo- and 3-bromophenylthiourea derivatives of 4-azatricyclo[5.2.1.0 2,6]dec-8-ene-3,5-dione were poorly active in comparison with the 4-chloro-3-nitrophenyl thioureas published in this paper [6]. The only exception was the 4-bromophenylthiourea with an azatricyclodione terminal fragment, which was four times more active against standard Staphylococcal isolates than its 3-chloro-4-fluorophenylthiourea analog (**3**). To conclude, derivatives of substituted phenylthioureas, mainly 3-trifluoromethylphenylthiourea complexes, revealed higher antimicrobial activity in comparison with coordinates of cyclic imides.

## 3. Materials and Methods

### 3.1. General Procedure

All chemicals were of analytical grade and were purchased from Sigma-Aldrich. The melting point (m.p.) was determined on a Boetius (HMK65/ 1360) microscope. Elemental analysis of all complexes was carried out using elemental analyzer CHNS (Vario Micro Cube) with an electronic microbalance. The copper content for complex **5** was determined using the energy dispersive X-ray fluorescence spectrometer EDX-7000 from Shimadzu.

#### 3.1.1. Synthesis of Cu (II) Complexes of 3-(4-Chloro-3-nitrophenyl)thiourea (**1**‒**8**)

The appropriate 3-(4-chloro-3-nitrophenyl)thiourea **L1**–**L8** (1 mmol) was stirred in dimethylformamide (DMF) (2 mL) until its dissolution. Next, anhydrous copper (II) chloride (1 mmol) was added to the solution. After stirring for 6 h, the solvent was evaporated. The solid residue was collected, washed several times with cold distilled water and dried in vacuo over anhydrous calcium chloride at room temperature to yield complexes **1**–**8**.

1. Copper (II) complex with 1-(2-bromophenyl)-3-(4-chloro-3-nitrophenyl)thiourea.

Yield 53%; dark brown solid; m.p. 93–95 °C; Anal. Calc for Cu(L1)_2_·0.75DMF·0.5H_2_O; Calc. C 37.76, H 2.50, N 10.52, Found C 37.92, H 2.28, N 10.57 (%).

2. Copper (II) complex with 1-(3-bromophenyl)-3-(4-chloro-3-nitrophenyl)thiourea.

Yield 61%; dark brown solid; m.p. 80–82 °C; Anal. Calc for Cu(L2)_2_·0.25 DMF·1.25 H2O, Calc. C 36.69, H 2.23, N 10.00, Found C 36.28, H 2.39, N 10.40 (%).

3. Copper (II) complex with 1-(4-bromophenyl)-3-(4-chloro-3-nitrophenyl)thiourea.

Yield 58%; dark brown solid; m.p. 88–90 °C; Anal. Calc for Cu(L3)_2_·0.75 DMF·0.5 H_2_O, Calc. C 37.76, H 2.50, N 10.52, Found C 38.04, H 2.24, N 10.30 (%).

4. Copper (II) complex with 1-(3-chloro-4-fluorophenyl)-3-(4-chloro-3-nitrophenyl)thiourea.

Yield 55%; dark brown solid; m.p. 95–97 °C; Anal. Calc for Cu(L4)_2_·0.75 DMF·0.5 H_2_O, Calc. C 40.12, H 2.41, N 11.18, Found C 40.26, H 2.18, N 11.30 (%).

5. Copper (II) complex with 1-(4-chloro-3-nitrophenyl)-3-(3,4-dichlorophenyl)thiourea.

Yield 57%; brown solid; m.p. 76–78 °C; Anal. Calc for Cu(L5)_2_·3.25H_2_O, Calc. C 35.76, H 2.37, N 9.62, Cu 7.28, Found C 35.76, H 2.20, N 9.88, Cu 7.74 (%).

6. Copper (II) complex with 1,3-bis(4-chloro-3-nitrophenyl)thiourea.

Yield 45%; light yellow solid; m.p. 107–108 °C; Anal. Calc for Cu(L6)_2_ ·0.75 DMF·0.75 H_2_O, Calc. C 37.52, H 2.31, N 13.55, Found C 37.41, H 2.22, N 13.41 (%).

7. Copper (II) complex with 1-(2-fluorophenyl)-3-(4-chloro-3-nitrophenyl)thiourea.

Yield 57%; yellow-green solid; m.p. 97–99 °C; Anal. Calc for Cu(L7)_2_·0.5 DMF·0.25 H_2_O, Calc. C 43.8, H 2.67, N 12.07, Found C 43.82, H 2.47, N 12.22 (%).

8. Copper (II) complex with 1-(4-iodophenyl)-3-(4-chloro-3-nitrophenyl)thiourea.

Yield 47%; orange brown solid; m.p. 99–101 °C; Anal. Calc for Cu(L8)_2_ ·3.5H_2_O, Calc. C 31.48, H 2.32, N 8.47, Found C 31.20, H 2.08, N 8.50 (%).

#### 3.1.2. Instrumentation

Infrared spectra were performed on Nicolet iS5 FTIR spectrometer (Thermo Scientific) with diamond ATR sample accessory. The complexes **1**–**8** as well as organic ligands **L1**–**L8** were recorded in the range of 400–4000 cm^−1^. The solid state electronic reflectance spectra of complexes **1**–**8** were collected on SHIMADZU UV-2600 spectrophotometer with UV-2600Plus Integrating Sphere in the range of 220–1400 nm. Electron paramagnetic resonance (EPR) spectra were recorded with a Bruker ELEXSYS-E580 X-band spectrometer (100 kHz field modulation). The microwave power of 5 mW and the modulation amplitude of 0.1–0.5 mT were applied. Prior, the measurements of the samples were dissolved in a mixture of ethanol–toluene solvent (1:1 *v*/*v*) to form solution of ca. 0.1 mM. The EPR measurements of frozen solutions were carried out at 77 K. The EPR parameters of the copper complexes were determined by computer simulation of the experimental spectra using the EPRsim32 package [60].

### 3.2. Cell Culture

The human primary (SW480), metastatic (SW620) colon cancer, metastatic prostate cancer (PC3) and human immortal keratinocyte (HaCaT) cell lines were purchased from the American Type Culture Collection (ATCC, Rockville, USA). The cells were cultured in medium according to protocols (MEM for SW480 and SW620, RPMI 1640 for PC3 and DMEM for HaCaT cells) supplemented with 10% fetal bovine serum (FBS), penicillin (100 U/mL) and streptomycin (100 μg/mL) and cultured in 37 °C/ 5% CO_2_ humidified incubator. The cells were cultured until appropriate confluence was achieved (80–90%). Next, they were harvested by treatment with 0.25% trypsin (Gibco Life Technologies, USA) and used for studies.

### 3.3. MTT Assay

To determine IC_50_ of the thiourea complexes, cells were seeded in 96-well plates (1 × 10^4^ cells per well) and treated for 72 h with different concentrations of compounds. Cells without studied compounds in medium were used as a control.

The cell viability was assessed by determination of MTT salt (3-(4,5-dimethylthiazol-2-yl)-2,5-diphenyltetrazolium bromide) conversion by mitochondrial dehydrogenase. MTT assay was performed as previously described [61]. Experiments were repeated three times. Cell viability was presented as a percentage of MTT reduction in the treated cells versus the control cells. Number of viable cells cultured without studied compounds was assumed to be 100%. Decreased relative MTT level means decreased cell viability. Thiourea complexes with the highest cytotoxic potential assessed by MTT determination (with the lowest IC_50_) were chosen for subsequent assessments of cytotoxicity mechanisms.

### 3.4. LDH Assay

The presence of lactate dehydrogenase (LDH) in culture medium indicates a disturbance of the integrity of the cellular membrane. The LDH activity was performed after 72 h incubation of cells (1 × 10^4^ cells per well) in 96-well plates with selected compounds according to manufacturer’s protocol (Roche Diagnostics, Germany), as was described by Chrzanowska et al. [61]. Compound mediated cytotoxicity was determined using equation: ((A test sample−A low control)/ (A high control−A low control)) × 100% (A-absorbance), where “low control” means cells in medium with 2% FBS without tested compounds, and “high control” means cells incubated in medium with 2% FBS and 1% Triton X-100 (100% LDH release). The cytotoxicity was expressed as percentage of LDH release as compared with the maximum release of LDH from Triton-X-100-treated cells.

### 3.5. Annexin V Binding Assay

The cells were cultured and harvested under the conditions described in the cell culture section. Then, they were seeded in six-well plates (2 × 10^5^ cells per well) and treated with selected thioureas complexes at their IC_50_ concentration for 72 h. The effect of these compounds on the process of early and late apoptosis and necrosis was determined, as described previously [61], by dual staining with annexin V-FITC and propidium iodide according to manufacturer’s protocol (Becton Dickinson). The cells that were annexin V-FITC positive and PI-negative were identified as early apoptotic and annexin V-FITC and PI-positive as late apoptotic or necrotic.

### 3.6. Il-6 Level Assay

IL-6 concentration at all studied cancer cells and normal HaCaT cell lines was measured by ELISA kit (Diaclon SAS Besancon Cedex, France). Cells were seeded in twelve-well plates (1 × 10^5^ cells per well) and treated with IC_50_ concentration of selected studied complexes for 72 h. IL-6 in cell culture supernatant was measured using enzyme-linked immunosorbent assay in accordance with the manufacturer’s protocol.

### 3.7. LC-MS Proteome Analysis

Enzymes involved in oxidoreductive potential were analyzed in the cell lysates obtained after treatment cells with selected conjugates for 24 h. Cells were washed with phosphate-buffered saline (PBS) and harvested, then centrifuged at 1000× *g* for 10 min. Then, lysis buffer (containing protease inhibitor, 1% RIPA Lysis and Extraction Buffer (ThermoFisher)) and cold PBS were added, and samples were sonicated three times in ice bath. Next, the cell lysates were centrifuged at 14,000× *g* at 4 °C for 15 min, and then supernatants were stored at 70 °C before use. Protein concentration was measured by the Bradford method.

Normalized protein concentrations (5 µg) from cell lysate were precipitated by ice cold (–20 °C) acetonitrile (ACN, Merck, in ratio 1:4). Then, samples were centrifuged (–9 °C, 30 min., 18,000× *g*), the supernatant was discarded and ACN excess was evaporated using a vacuum centrifuge (5 min., room temp.). Protein pellet was dissolved in 40 mM ammonium bicarbonate. An amount of 500 mM dithiothreitol (DTT, with final concentration 20 mM) and 1 M iodoacetamide (IAA, with final concentration 40 mM) were used for reduction and alkylation processes. After 16 h of incubation in 37 °C with Trypsin Gold (Promega), digested protein samples were diluted with 0.1% formic acid (ThermoFisher) and centrifuged (+2 °C, 30 min, 18,000× *g*).

LC–MS analysis was carried out with the use of nanoUHPLC (nanoElute, Bruker) coupled by CaptiveSpray (Bruker) to ESI-Q-TOF mass spectrometer (Compact, Bruker). Two-column separation method was used, i.e., pre-column (300 µm × 5 mm, C18 PepMap 100, 5 µm, 100Å, Thermo Scientific) and Aurora separation column with CSI fitting (75 µm × 250 mm, C18 1.6 µm) in gradient 2% B to 35% B in 90 min with the 300 nL/min flow rate. Mobile phases (A) 0.1% formic acid in water and (B) 0.1% formic acid in ACN were used.

Sample ionizations were performed at a gas flow of 3.0 L/min, temperature of 150 °C and voltage of the capillary at 1600 V. The quadrupole energy was fixed to 5.0 eV and collision chamber energy 7.0 eV, with an ion transfer time of 90 µs. The ions were analyzed in the positive polarity mode in the range 150–2200 *m*/*z*, with the acquisition frequency of the 1 Hz spectrum, as well as with the autoMS/MS system.

The collected spectra were analyzed and calibrated using DataAnalysis software (Bruker) and then identified in ProteinScape (Bruker) by the MASCOT server. Protein identification was conducted using the online SwissProt and NCBIprot databases, and their references and biological significance were identified using Reactome.org, String.org and KEGG.

### 3.8. In Vitro Evaluation of Antimicrobial Activity

The antimicrobial activity of the compounds was tested on Gram-positive bacteria (*Staphylococcus aureus* NCTC 4163, *Staphylococcus aureus* ATCC 25923, *Staphylococcus aureus* ATCC 6538, *Staphylococcus aureus* ATCC 29213, *Staphylococcus epidermidis* ATCC 12228, *Staphylococcus epidermidis* ATCC 35984), Gram-negative rods (*Escherichia coli* ATCC 10538, *Escherichia coli* ATCC 25922, *Pseudomonas aeruginosa* ATCC 15442, *Pseudomonas aeruginosa* ATCC 27863) and yeasts (*Candida albicans* ATCC 10231, *Candida albicans* ATCC 90028, *Candida parapsilosis* ATCC 22019). Hospital methicillin-resistant strains of *Staphylococcus aureus* and *Staphylococcus epidermidis* were obtained from the collection of the Department of Pharmaceutical Microbiology, Medical University of Warsaw, Poland.

Antibacterial activity was examined by the disc-diffusion method under standard conditions using Mueller–Hinton II agar medium (Becton Dickinson) according to CLSI (previously NCCLS) guidelines [62]. Antifungal activities were assessed using Mueller–Hinton agar + 2% glucose and 0.5 µg/mL methylene blue dye medium [63]. Sterile filter paper discs (9 mm diameter, Whatman Number 3 chromatography paper) were dripped with tested compound solutions (in dimethylsulfoxide, DMSO) to load 400 μg of a given compound per disc. Dry discs were placed on the surface of appropriate agar medium. The results (diameter of the growth inhibition zone) were read after 18 h of incubation at 35 °C. Minimal inhibitory concentration (MIC) was tested by the twofold serial microdilution method (in 96-well microtiter plates) using Mueller–Hinton broth medium (Beckton Dickinson) for bacteria or RPMI-1640 medium for *Candida* species, according to CLSI guidelines [64,65]. The stock solution of tested agent was prepared in DMSO and diluted in sterile water. Concentrations of tested agents ranged from 0.125 to 512 µg/mL. The final inoculum of all studied microorganisms was 10^5^ CFU/ mL^−1^ (colony forming units per ml). Minimal inhibitory concentrations (the lowest concentration of a tested agent that prevents visible growth of a microorganism) were read after 18 h (bacteria) or 24 h (yeasts) of incubation at 35 °C.

### 3.9. Genotoxicity Studies

DNA-damaging activity of compounds was tested by rec-assay using two genetically modified *Bacillus subtilis* strains: M45 (rec^−^) and H17 (rec^+^) [66,67]. Tested compounds were dissolved in DMSO, and 10 µL of each solution was dripped onto sterile cotton discs (Rotilabo) to load 256 µg of a given compound per 9 mm disc. Discs were placed on the surface nutrient agar plates (Difco), inoculated with 100 µL of bacterial overnight culture and incubated 24 h at 35 °C. After incubation, the growth inhibition zones were measured. 4-nitroquinoline N-oxide (NOQ) was used as reference genotoxin (concentration 2 µg per disc). Results of the genotoxicity test were estimated after 18 h of incubation at 35 °C by comparing the diameter of the inhibition zone on the *B. subtilis* M45 (rec^−^) strain with that on the *B. subtilis* H17 (rec^+^) strain.

### 3.10. Statistical Analyses

Statistical analyses were performed using GraphPad Prism 9 software (GraphPad Software). Statistical significance was assessed by ANOVA with Dunnett’s post hoc test. P values below 0.05 were considered statistically significant. Data were presented as the mean ± SD from at least three independent experiments.

## 4. Conclusions

To conclude, we have presented the cytotoxic influences of four-coordinate Cu (II) complexes with 3-(4-chloro-3-nitrophenyl)thiourea derivatives on a panel of human cancer and normal cell lines, as well as on bacterial isolates. In contrast to initial ligands, the complexation with metal ions has revealed the cytotoxic profile of synthesized compounds towards tumor cells and to a lesser extent to bacterial strains. Diversity of a type, a position and quantity of halogen substituents on the phenyl ring of the thiourea branch allowed for the examination of the impact of the structure of synthesized complexes on their bioactivity. Studied coordination compounds did not express cytotoxic effects in normal cells (HaCaT) at clinically achievable concentrations, and they were also proven to be non-genotoxic. The most active halogen phenylthiourea complexes (**1**, **3**, **7**, **8**) showed stronger anticancer potential against PC3 compared to colon cancer cell lines. They were also more effective than the tested disubstituted derivatives (**4**–**6**). Compound **8** achieved the highest percentage of LDH release from PC3 and SW480 cells. Studied complexes, especially **3** and **8**, induced early apoptosis in the above-mentioned pathological cells. Additionally, all coordination compounds, with emphasis on derivatives **1** and **8**, reduced the secretion of IL-6 by tumor cell lines. Their interleukin-inhibitory properties in the selected cells were equally as strong as doxorubicin. Moreover, new compounds were active against selected strains of Staphylococci of clinical importance, being up to 2–64 times more potent in comparison to the reference antibiotic, ciprofloxacin. Our preliminary studies also showed the general tendency of tested compounds to disturb the antioxidant and detoxifying systems in cancer cells, especially evident for complexes **3**, **7** and **8**. This is an initial signal that this may be one of the cytotoxicity mechanisms. Such a diminishing effect of compounds on the antioxidant defense of cancer cells may support the action of other pro-oxidative agents, including drug resistance as well as support radiotherapy treatment.

## Figures and Tables

**Table 1:** Characteristic infrared absorption bands of N−H, NO_2_, C=S and C−X (X = Br, Cl, F, I) groups. Band position in cm^−1^, intensity: w is weak, m is medium, s is strong, vs is very strong, sh is shoulder, ^as^ is asymmetric, ^s^ is symmetric, * is broad band.

Compound	υN−H	υ^as^NO_2_	υ^s^NO_2_	υC=S	υC−X
**1**	3470 w, 3372 m	1529 m *	1347 m *	1301 m, 782 w	1047 w
**L1**	3340 w, 3209 m	1537 sh, 1526 vs	1347 sh	1332 m, 832 m	1044 m
**2**	3444 w, 3395 w	1531 m *	1344 m *	1306 w, 776 w	1070 w
**L2**	3334 sh, 3250 m	1547 m, 1526 m	1352 m, 1333 s	1332 m, 834 w	1067 m
**3**	3448 w, 3333 w	1530 m *	1351 m *	1305 w, 774 sh	1071 w
**L3**	3361 w, 3317 s	1537 sh, 1525 s	1355 m *	−, 839 w	1070 m
**4**	3449 w, 3335 m	1531vs *	1344 m *	1311 m, 781 w	1051 m
**L4**	3365 sh, 3244 s	1546 m, 1525 s	1353 m, 1335 s	1335 m, 840 m	1049 m
**5**	3449 w, 3333 m	1529 vs *	1344 m *	1300 m, 779 sh	1050 m
**L5**	3348 sh, 3243 s	1539 vs, 1509 sh	1347 sh, 1331 s	1331 m, 835 m	1050 m
**6**	3449 w, 3340 m	1539 sh, 1519 vs	1341 m *	1313 m, 784 w	1044 m
**L6**	3327 s, 3243 m	1542 m, 1515 s	1353 w, 1333 m	1333 m, 837 sh	1047 m
**7**	3446 w, 3341 m	1532 m *	1346 m *	1313 m, 785 w	1236 w
**L7**	3325 s, 3242 m	1539 s, 1518 vs	1356 s, 1335 s	1335 m, 854 w	1232 w
**8**	3448 w, 3336 w	1532 m *	1350 m *	1307 w, 771 w	1057 w
**L8**	3364 s, 3314 m	1538 s, 1525 s	1350 s *	1331 sh, 833 sh	1059 m

**Table 2:** Cytotoxic activity (IC_50_, µM) of studied compounds estimated by the MTT assay ^a^.

Compound		Cancer Cells						Normal Cells
		SW480 ^d^		SW620 ^e^		PC3 ^f^		HaCaT ^g^
	R	IC_50_ ^b^	SI ^c^	IC_50_	SI	IC_50_	SI	IC_50_
**1**	2-Br-Ph	4.7 ± 0.3	23.2	3.3 ± 0.2	33.2	9.7 ± 0.1	11.5	109.6 ± 3.4
**2**	3-Br-Ph	24.3 ± 2.6	6.8	22.3 ± 1.8	7.5	19.2 ± 2.2	8.7	167.2 ± 2.3
**3**	4-Br-Ph	11.9 ± 2.1	8.6	19.2 ± 2.3	5.3	8.8 ± 0.8	11.7	103.2 ± 3.2
**4**	3-Cl,4-F-Ph	19.2 ± 1.1	6.3	21.6 ± 2.9	5.7	23.2 ± 1.6	5.2	120.9 ± 5.2
**5**	3-Cl,4-Cl-Ph	20.6 ± 2.1	8.1	10.8 ± 2.6	15.2	11.4 ± 2.4	14.4	164.9 ± 4.7
**6**	3-NO_2_,4-Cl-Ph	26.8 ± 2.3	4.6	21.5 ± 1.4	5.7	20.3 ± 3.1	6.1	123.2 ± 2.1
**7**	2-F-Ph	15.5 ± 2.6	8.9	9.1 ± 0.8	12.8	10.8 ± 1.3	5.5	138.3 ± 4.4
**8**	4-I-Ph	3.9 ± 0.8	26.3	17.8 ± 1.3	5.7	4.3 ± 0.5	23.8	102.7 ± 3.4
Doxorubicin ^h^		0.75 ± 0.1	0.4	0.26 ± 0.1	1.1	0.31 ± 0.1	0.9	0.29 ± 0.1
Cisplatin ^i^		10.4 ± 0.9	0.6	6.7 ± 1.1	0.9	13.2 ± 2.1	0.5	6.3 ± 0.7
CuCl_2_ ^j^		109.4 ± 7.6	1.0	96.3 ± 5.2	1.2	106.5 ± 6.3	1.1	114.3 ± 4.8

^a^ MTT: 3-(4,5-dimethylthiazol-2-yl)-2,5-diphenyltetrazolium bromide. Data are expressed as mean standard deviation (SD); ^b^ IC_50_ (half-maximal inhibitory concentration, µM): the concentration of the compound that corresponds to a 50% growth inhibition of the cell line (as compared to the control) after culturing the cells for 72 h with the individual compound; ^c^ the SI (selectivity index) was calculated using the formula SI = IC_50_ for normal cell line/IC_50_ cancer cell line; ^d^ human primary colon cancer (SW480); ^e^ human metastatic colon cancer (SW620); ^f^ human metastatic prostate cancer (PC3); ^g^ human immortal keratinocyte cell line from adult human skin (HaCaT); ^h,i,j^ the reference compounds.

**Table 3:** Liquid chromatography–mass spectrometry (LC–MS) proteome analysis provided in the SW480, SW620 and PC3 cells treated for 24 h with IC_50_ concentrations of complexes **1**, **3**, **7** and **8**. Protein intensities were expressed as percentage of a control.

Protein Intensity, %																
Accession	Name of Enzyme	SW480					SW620					PC3				
		Control	1	3	7	8	Control	1	3	7	8	Control	1	3	7	8
GSTA1_HUMAN	Glutathione S-transferase A1 OS = Homo sapiens OX = 9606 GN = GSTA1 PE = 1 SV = 3	100	78.3	86.6	75.6	91.6	100	50.2	45.2	36.4	54.3	100	84.4	70.6	79.2	73.5
GSTO1_HUMAN	Glutathione S-transferase omega-1 OS = Homo sapiens OX = 9606 GN = GSTO1 PE = 1 SV = 2	100	70.9	66.0	45.1	52.4	100	85.5	77.0	52.5	65.0	100	65.0	64.0	68.6	31.6
GSTP1_HUMAN	Glutathione S-transferase P OS = Homo sapiens OX = 9606 GN = GSTP1 PE = 1 SV = 2	100	84.8	75.9	80.9	83.0	100	74.4	64.5	67.3	57.5	100	95.0	51.1	35.8	64.1
GSHR_HUMAN	Glutathione reductase, mitochondrial OS = Homo sapiens OX = 9606 GN = GSR PE = 1 SV = 2	100	58.1	63.1	91.7	74.3	100	69.1	79.2	67.3	79.3	100	71.1	58.9	61.2	68.5
SODC_HUMAN	Superoxide dismutase [Cu-Zn] OS = Homo sapiens OX = 9606 GN = SOD1 PE = 1 SV = 2	100	68.2	75.3	40.6	88.2	100	72.7	78.2	44.7	64.5	100	58.5	30.9	15.4	33.0
SODM_HUMAN	Superoxide dismutase [Mn], mitochondrial OS = Homo sapiens OX = 9606 GN = SOD2 PE = 1 SV = 3	100	67.8	77.7	65.1	94.3	100	88.8	77.1	45.3	92.7	100	29.1	31.2	25.2	29.4
PRDX1_HUMAN	Peroxiredoxin-1 OS = Homo sapiens OX = 9606 GN = PRDX1 PE = 1 SV = 1	100	95.9	88.5	71.6	84.4	100	96.1	89.0	83.2	93.5	100	73.9	47.8	46.5	52.3
PRDX2_HUMAN	Peroxiredoxin-2 OS = Homo sapiens OX = 9606 GN = PRDX2 PE = 1 SV = 5	100	95.5	93.7	69.4	78.3	100	92.1	71.6	58.6	88.6	100	42.3	47.3	32.2	39.9
PRDX4_HUMAN	Peroxiredoxin-4 OS = Homo sapiens OX = 9606 GN = PRDX4 PE = 1 SV = 1	100	67.6	60.1	58.3	78.2	100	79.4	76.4	73.0	93.9	100	72.6	61.1	35.7	30.8
PRDX5_HUMAN	Peroxiredoxin-5, mitochondrial OS = Homo sapiens OX = 9606 GN = PRDX5 PE = 1 SV = 4	100	77.3	84.8	91.0	81.7	100	88.3	82.4	81.4	74.4	100	29.3	40.9	36.7	42.0
PRDX6_HUMAN	Peroxiredoxin-6 OS = Homo sapiens OX = 9606 GN = PRDX6 PE = 1 SV = 3	100	75.1	81.3	68.3	71.1	100	83.2	66.4	67.5	77.6	100	66.6	71.1	35.0	53.7
PRDX3_HUMAN	Thioredoxin-dependent peroxide reductase, mitochondrial OS = Homo sapiens OX = 9606 GN = PRDX3 PE = 1 SV = 3	100	95.2	85.4	90.4	96.3	100	69.9	67.5	61.2	95.2	100	69.5	57.3	12.4	24.8

**Table 4:** In vitro activity of complexes **1**–**8** against standard bacterial and fungal strains—minimal inhibitory concentrations (MIC, μg/mL).

Strain	1	2	3	4	5	6	7	8	Ref. *	Ref. **
*S. aureus* NCTC 4163	8	16	8	4	32	8	32	4	0.25	-
*S. aureus* ATCC 25923	8	16	8	4	32	8	32	4	0.5	-
*S. aureus* ATCC 6538	8	16	8	4	32	8	32	4	0.25	-
*S. aureus* ATCC 29213	8	16	16	4	32	8	32	4	0.25	-
*S. epidermidis* ATCC 12228	16	16	16	4	32	8	32	8	0.25	-
*S. epidermidis* ATCC 35984	8	16	16	4	32	8	32	4	≤0.125	-
*E. coli* NCTC 10538	128	128	128	128	128	128	128	128	≤0.125	-
*E. coli* ATCC 25922	256	128	>256	128	128	128	256	128	≤0.125	-
*P. aeruginosa* ATCC 15442	128	128	128	128	128	128	>256	128	0.5	-
*P. aeruginosa* ATCC 27853	128	128	128	128	128	128	128	128	0.5	-
*C. albicans* ATCC 10231	≥256	64	64	128	128	64	128	64	-	0.5
*C. albicans* ATCC 90028	≥256	64	64	128	128	64	128	128	-	0.5
*C. parapsilosis* ATCC 22019	≥256	64	64	64	64	64	128	64	-	0.5

Ref. *—Ciprofloxacin, Ref. **—Fluconazole.

**Table 5:** In vitro activity of complexes **1**–**8** against hospital methicillin-resistant strains of *Staphylococcus aureus* (MRSA) and *Staphylococcus epidermidis* (MRSE)—minimal inhibitory concentrations (MIC, µg/mL).

Strain	1	2	3	4	5	6	7	8	Ref. *
*S. aureus* 498	8	8	4	4	16	32	4	8	0.5
*S. aureus* 537	8	8	4	4	16	32	8	8	256
*S. aureus* 567	8	8	4	4	16	32	4	8	0.5
*S. aureus* 568	8	8	4	4	16	32	8	8	0.5
*S. aureus* 573	8	8	4	4	16	32	8	8	128
*S. aureus* 585	8	8	4	4	16	32	8	8	256
*S. aureus* 586	4	8	4	4	16	32	8	8	0.5
*S. aureus* 495	8	8	4	4	16	32	8	4	0.5
*S. aureus* 496	8	8	4	8	32	32	8	8	0.25
*S. aureus* 497	8	8	4	8	16	32	8	8	256
*S. aureus* 514	8	8	4	4	32	32	8	4	128
*S. aureus* 522	8	8	4	4	16	32	8	4	256
*S. aureus* 572	8	8	4	4	16	32	4	4	256
*S. aureus* 481	8	8	4	4	16	32	4	4	256
*S. epidermidis* 420	4	8	8	4	32	64	8	8	0.5
*S. epidermidis* 423	8	8	4	4	32	64	8	8	0.5
*S. epidermidis* 424	8	8	4	8	32	64	8	8	16
*S. epidermidis* 469	8	8	4	4	32	64	8	8	0.5
*S. epidermidis* 471	8	8	8	8	32	64	8	8	32
*S. epidermidis* 510	8	8	4	8	16	64	8	8	0.5
*S. epidermidis* 511	8	8	4	4	32	64	8	8	32
*S. epidermidis* 515	4	8	8	4	32	64	8	8	32
*S. epidermidis* 431	8	8	4	8	16	64	8	8	8
*S. epidermidis* 432	8	8	4	8	32	64	8	8	64
*S. epidermidis* 433	4	8	4	4	32	64	8	4	64
*S. epidermidis* 435	8	8	4	8	32	128	8	8	0.25
*S. epidermidis* 436	8	8	4	8	32	128	8	8	≤ 0.125
*S. epidermidis* 437	8	8	8	8	32	128	8	8	0.5
*S. epidermidis* 438	8	8	4	8	32	128	8	8	≤ 0.125
*S. epidermidis* 513	8	8	4	8	16	64	8	8	0.5

Ref. *—Ciprofloxacin.

## Supplementary Materials

The following are available online at https://www.mdpi.com/article/10.3390/ijms222111415/s1.
