# Supplementary material for: The Cytotoxic Effect of Copper (II) Complexes with Halogenated 1,3-Disubstituted Arylthioureas on Cancer and Bacterial Cells
Source: Int J Mol Sci. 2021 Oct 22;22(21):11415. doi: 10.3390/ijms222111415 (PMC8584173; doi:10.3390/ijms222111415)
Supplement: Supplementary file 1 [file ijms-22-11415-s001.zip › ijms-1400594-supplementary.pdf]

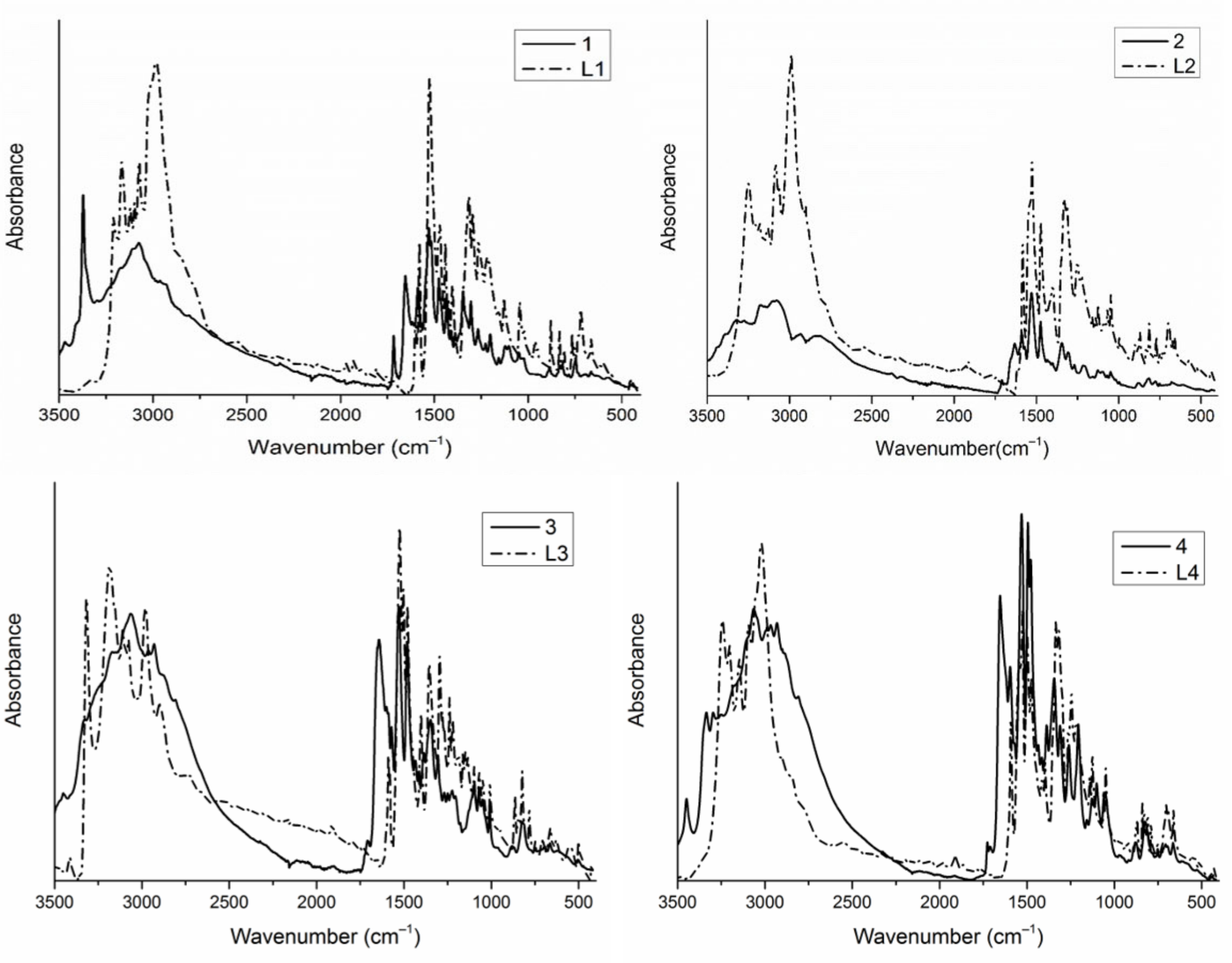
1
L1
Absorbance
3500
3000
2500
2000
1500
1000
500
Wavenumber (cm−1)
2
L2
Absorbance
Wavenumber(cm−1)
3
L3
Absorbance
Wavenumber (cm−1)
4
L4
Absorbance
Wavenumber (cm−1)

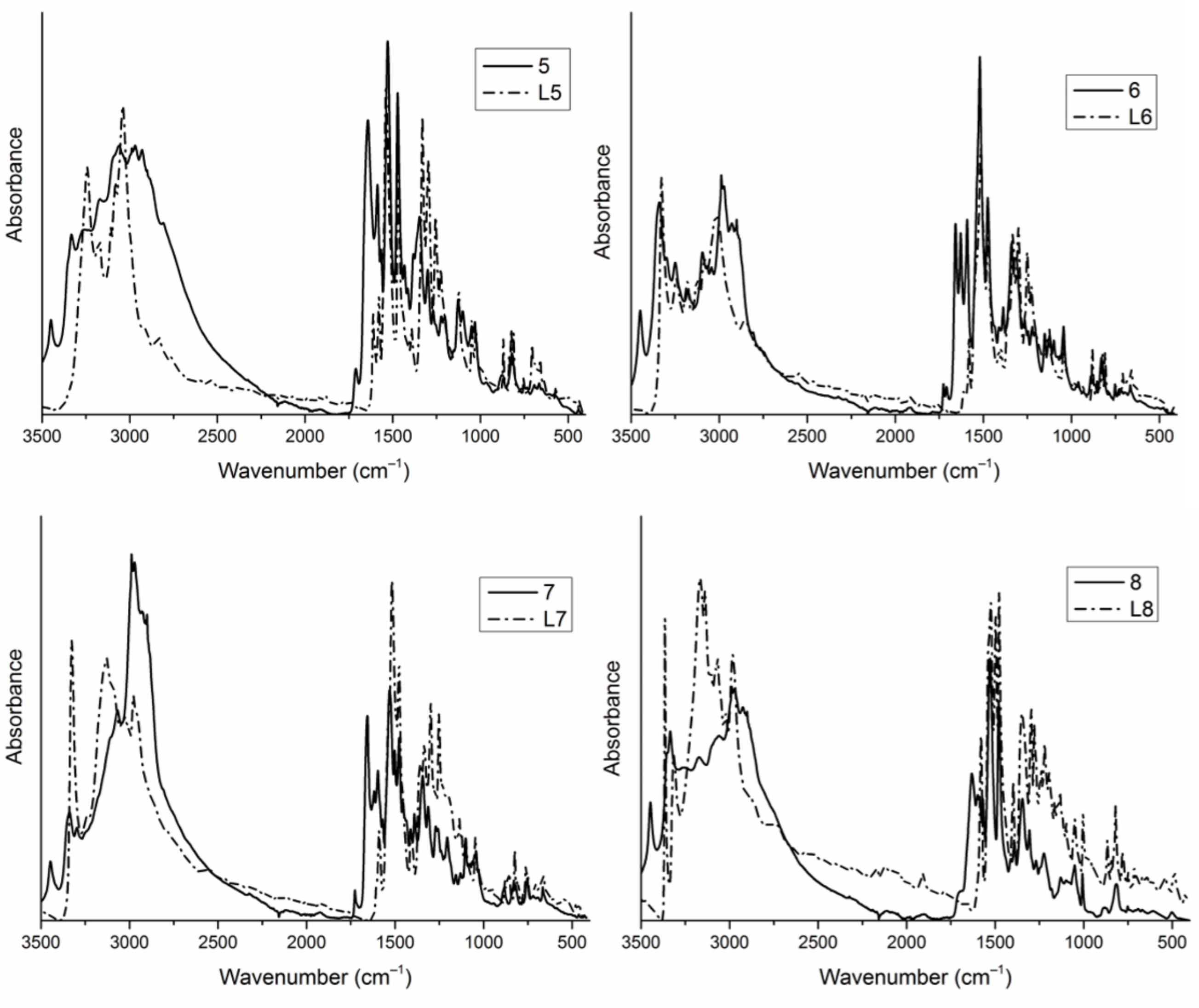

Figure S1. ATR-FTIR spectra of Cu(II) complexes (solid line) and parent ligands (dash dot line).

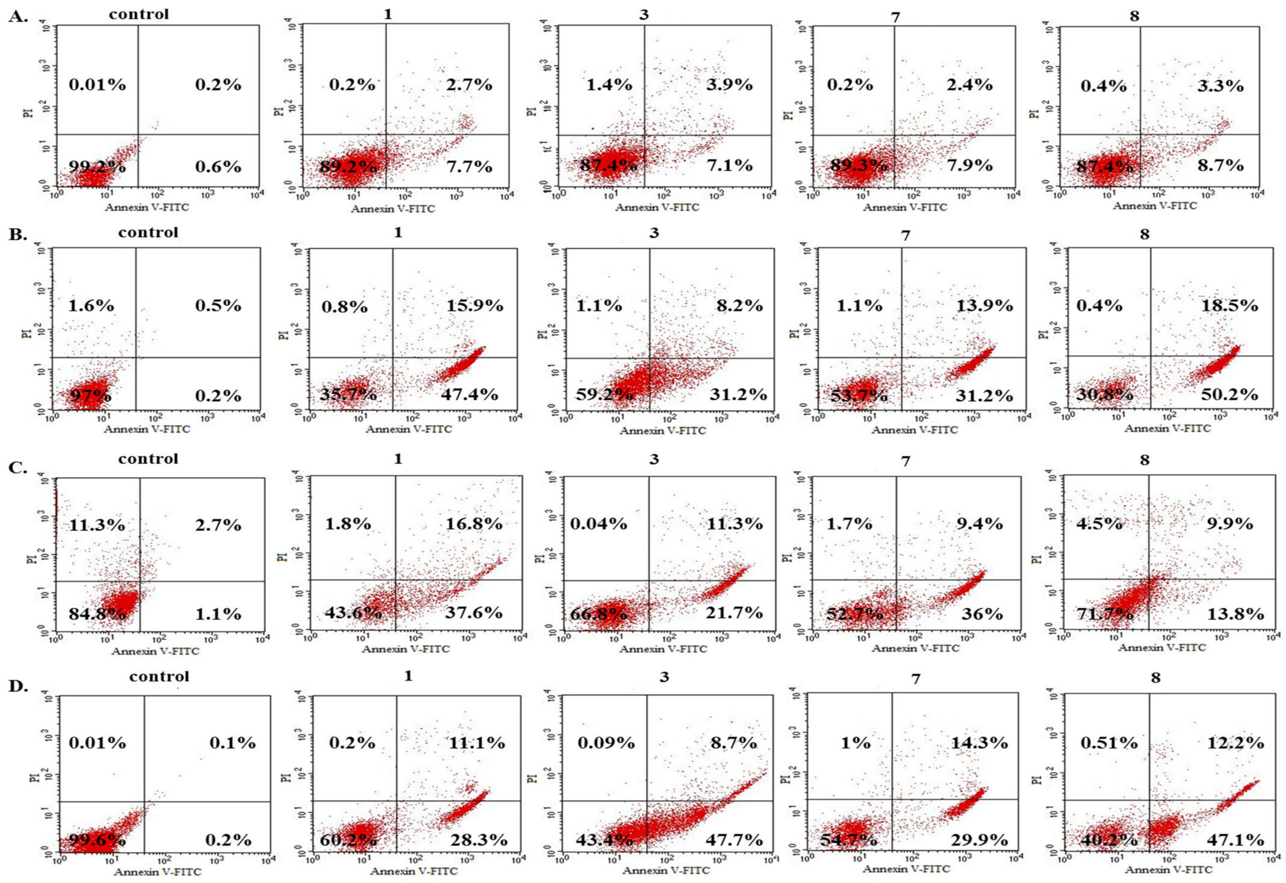

Figure S2. The effect of compounds **1, 3, 7** and **8** on early and late apoptosis or necrosis in (**A.**) HaCaT, (**B.**) SW480, (**C.**) SW620 and (**D.**) PC3 cells detected by flow cytometry. Cells were incubated for 72 h with compounds. Diagrams show representative experiments. The lower right quadrant shows early apoptotic cells. The upper right and upper left quadrants represent late stage of apoptotic or necrotic cells.

Table S1. LC-MS proteome analysis provided in the SW480, SW620 and PC3 cells treated for 24h with IC_{50} concentrations of complexes **1, 3, 7** and **8.** Protein intensities were expressed as a mean from three independent experiments.

**Accession**	**Name**	**SW480**					**SW620**					**PC3**				
		**Control**	**1**	**3**	**7**	**8**	**Control**	**1**	**3**	**7**	**8**	**Control**	**1**	**3**	**7**	8
GSTA1_HUMAN	Glutathione S-transferase A1 OS=Homo sapiens OX=9606 GN=GSTA1 PE=1 SV=3	**413234**	323678	357876	312455	378434	**91232**	45787	41221	33213	49567	**627640**	529646	443212	497323	461232
GSTO1_HUMAN	Glutathione S-transferase omega-1 OS=Homo sapiens OX=9606 GN=GSTO1 PE=1 SV=2	**298234**	211398	196867	134565	156145	**232112**	198345	178657	121889	150987	**341337**	221750	218400	234323	107996
GSTP1_HUMAN	Glutathione S-transferase P OS=Homo sapiens OX=9606 GN=GSTP1 PE=1 SV=2	**367245**	311345	278656	297141	304789	**345221**	256768	222545	232433	198656	**1425197**	1353319	728000	510193	914173
GSHR_HUMAN	Glutathione reductase, mitochondrial OS=Homo sapiens	**195334**	113567	123212	179090	145212	**162345**	112122	128575	109244	128676	**158064**	112362	93164	96786	108221

	OX=9606 GN=GSR PE=1 SV=2															
SODC_HUMAN	Superoxide dismutase [Cu-Zn] OS=Homo sapiens OX=9606 GN=SOD1 PE=1 SV=2	**1122335**	765868	845454	456223	989556	**1223356**	889767	956678	546788	789556	**1429442**	835640	441746	220268	471498
SODM_HUMAN	Superoxide dismutase [Mn], mitochondrial OS=Homo sapiens OX=9606 GN=SOD2 PE=1 SV=3	**789265**	534998	612990	513998	744634	**875342**	776998	675159	396766	812234	**577101**	168127	179840	145456	169600
PRDX1_HUMAN	Peroxiredoxin-1 OS=Homo sapiens OX=9606 GN=PRDX1 PE=1 SV=1	**31223231**	29933676	27629276	22345478	26367811	**33456762**	32167221	29767565	27845632	31277885	**24399792**	18022715	11654561	11354508	12771180
PRDX2_HUMAN	Peroxiredoxin-2 OS=Homo sapiens OX=9606 GN=PRDX2 PE=1 SV=5	**6546284**	6248567	6133778	4545432	5123436	**7216764**	6647586	5168773	4227345	6396714	**5454113**	2304572	2581892	1758727	2174218
PRDX3_HUMAN	Thioredoxin-dependent peroxide reductase, mitochondrial OS=Homo sapiens OX=9606 GN=PRDX3 PE=1 SV=3	**8121243**	7734523	6939454	7338476	7823234	**923454**	645744	623451	564878	878679	**764279**	531444	437643	94596	189285
PRDX4_HUMAN	Peroxiredoxin-4 OS=Homo sapiens OX=9606 GN=PRDX4 PE=1 SV=1	**6549871**	4428616	3935987	3820901	5124476	**5456223**	4334576	4167234	3982563	5123234	**2970723**	2155687	1813810	1061038	914499
PRDX5_HUMAN	Peroxiredoxin-5, mitochondrial OS=Homo sapiens OX=9606 GN=PRDX5 PE=1 SV=4	**1453456**	1123451	1232982	1322454	1187676	**1121232**	989873	923434	912343	834567	**952281**	278662	389694	349564	399555
PRDX6_HUMAN	Peroxiredoxin-6 OS=Homo sapiens OX=9606 GN=PRDX6 PE=1 SV=3	**8765578**	6578981	7122234	5987678	6234541	**9893937**	8234564	6567878	6678723	7678223	**5912981**	3936503	4206439	2070729	3177120

Table S2. Genotoxic activity of tested complexes – inhibition zone diameter (mm).

**Compound**	***Bacillus subtilis strain***	
	**H17**	**M45**
**1**	12	12
**2**	17	19
**3**	15	15
**4**	16	18
**5**	17	18
**6**	16	15

**7**	15	18
**8**	14	14
NOQ*	13	25

***NOQ** – 4-Nitroquinoline-N-oxide

DMF effect on cell lines

The cytotoxic effect of DMF towards cancer, normal and bacterial cells, as other popular solvents, was tested before by various scientific teams [1S-4S]. It seems to act on cells metabolism only when used in millimolar concentration, whereas IC_{50} of our active compounds was below 10 μM (and the highest IC_{50} found equaled 120 μM). As calculated, approximated concentration of DMF in a culture medium in MTT test, included with our complexes (for example complex 1, bounded with 0.75 mole of DMF) varied from 2 μM = 0.002 mM (SW 480 cells) to nearly 82 μM =0.08 mM (HaCaT cells). Concerning bacterial studies, DMF might be found in a medium at maximum concentration (equaled MIC = 4 μg/ml for the most active compounds) of 0.004 μM up to 0.12 μM. The effect of DMF on bioactivity in our tests is negligible, because we use micromolar, not millimolar amounts of substances. The impact of DMF on cell lines was also studied previously as follows:

1. effect on HaCaT cells and cancer cells [1]

In the experiments performed by Authors of that paper, DMF influenced HaCaT cells at concentration of 0.14 mg/ml (2 mM), and cancer cells applied at 1.8-9.5 mM. In our experiments, HaCaT cell were treated with 0.08 mM DMF (bounded with a complex 1) or maximum 0.1 mM for other complexes.

2. effect on colon cancer cells [2]

According to Authors, DMF exerted no cytotoxic effect towards colon cancer cells and inhibits cell proliferation by 40-50% at 80 μM (our complexes 1 and 8, at 4 μM caused apoptosis of SW480 cells in 47%). DMF at 100 μM did not increase LDH release from colon cancer cells, as compared to our derivative 8 (used at 60 μM, max. concentration of DMF 45 μM), which achieved 64% LDH release in PC3 cells, and 58% in SW480 cell lines.

3. effect on pancreatic cancer cells [3]

According to Authors, DMF caused 85% apoptosis of PC3 cells when applied at concentration of 200 μM, and PANC-1 in 30% (at 100 μM). Our complexes gave proapoptotic effect in their IC50 concentrations, i.e. 4-10 μM.

4. effect on bacterial cells [4]

In the experiments performed by Authors of that paper (Table 2), the number of living bacterial cells in the presence of DMF applied in 4.8% (v/v), i.e. about 60 mM, varied from 0.1 to 8.9%. Approximated concentration of DMF in our medium was only from 0.004 μM to 0.12 μM.

References

1. Ilieva, Y.; Dimitrova, L.; Zaharieva, M.M.; Kaleva, M.; Alov, P.; Tsakovska, I.; Pencheva, T.; Pencheva-El Tibi, I.; Najdenski, H.; Pajeva, I. Cytotoxicity and Microbicidal Activity of Commonly Used Organic Solvents: A Comparative Study and Application to a Standardized Extract from Vaccinium macrocarpon. *Toxics* **2021**, 9, 92. https://doi.org/10.3390/toxics9050092
2. Kaluzki, I.; Hailemariam-Jahn, T.; Doll, M.; Kaufmann, R.; Balermpas, P.; Zöller, N.; Kippenberger, S.; Meissner, M. Dimethylfumarate Inhibits Colorectal Carcinoma Cell Proliferation: Evidence for Cell Cycle Arrest, Apoptosis and Autophagy. *Cells* **2019**, 8, 1329. https://doi.org/10.3390/cells8111329
3. Chen, K.; Wu, S.; Ye, S.; Huang, H.; Zhou, Y.; Zhou, H.; Wu, S.; Mao, Y.; Shangguan, F.; Lan, L.; Chen, B. Dimethyl Fumarate Induces Metabolic Crisie to Suppress Pancreatic Carcinoma. *Front. Pharmacol.* **2021,** 12, 617714. https://doi.org/10.3389/fphar.2021.617714
4. Dyrda, G.; Boniewska-Bernacka, E.; Man, D.; Barchiewicz, K.; Słota, R. The effect of organic solvents on selected microorganisms and model liposome membrane. *Mol. Biol. Rep.* **2019,** 46(3), 3225–3232. https://doi.org/10.1007/s11033-019-04782-y
